# Fungal Contamination of Dairy Feed and Major Mycotoxin Transfer: A Risk Evaluation for Animal Exposure and Health

**DOI:** 10.3390/toxins18010042

**Published:** 2026-01-13

**Authors:** Ioana Poroșnicu, Luminița-Iuliana Ailincăi, Mădălina Alexandra Davidescu, Mihai Mareș

**Affiliations:** 1 Department of Public Health, Faculty of Veterinary Medicine, “Ion Ionescu de la Brad” Iasi University of Life Sciences, 700489 Iasi, Romania; ioana.porosnicu@yahoo.com (I.P.); mihai.mares@iuls.ro (M.M.); 2 Research and Development Station for Cattle Breeding Dancu, 707252 Iasi, Romania; 3 Department of Control and Expertise, Faculty of Food and Animal Sciences, “Ion Ionescu de la Brad” Iasi University of Life Sciences, 700489 Iasi, Romania; madalina.davidescu@iuls.ro

**Keywords:** mycotoxins, aflatoxin M1, dairy cattle, fungal ecology, feed contamination, *Aspergillus* spp., *Fusarium* spp.

## Abstract

This study was focused on the assessment of fungal occurrence, mycotoxin dynamics, aflatoxin carry-over, and associated biochemical responses in dairy cattle. Moisture emerged as the dominant factor for fungal communities, promoting the co-proliferation of fungal genera adapted to high water activity conditions (a_w_ > 0.90) and antagonism against xerotolerant and xerophilic species. *Aspergillus* spp. dominated dry substrates (a_w_ < 0.75), *Fusarium* spp. showed strong positive associations with high-moisture matrices (a_w_ > 0.90), and *Penicillium* spp. exhibited intermediate, substrate-dependent behavior. Mycotoxin levels fluctuated non-linearly, independently of fungal counts: ochratoxin A (OTA) concentrations in corn silage increased from approximately 12 μg/kg at the onset of the ensiling period to >240 μg/kg at silo opening, indicating dynamic mycotoxin accumulation during storage, while zearalenone (ZEA) oscillated from 40 to 170 µg/kg. Despite the variation in total aflatoxins (AFLA-T) across feed matrices, aflatoxin M1 (AFM1) in milk remained low (0.0020–0.0093 μg/kg), confirming limited carry-over. Serum biochemical parameters—alanine aminotransferase (ALT), aspartate aminotransferase (AST), gamma-glutamyl transferase (GGT), alkaline phosphatase (ALP), total bilirubin (BIL-T), total protein (PROT-T)—remained within physiological limits, yet multivariate analyses revealed metabolic modulation linked to aflatoxin exposure. AFM1 explained >7% of the variance in serum biochemical profiles according to PERMANOVA (*p* = 0.002), showed significant MANOVA effect (Pillai = 0.198), and displayed a significant canonical association (*p* < 10^−13^). Linear discriminant analysis further separated Normal vs. Borderline hepatic profiles, indicating subclinical physiological adaptation to chronic low-dose exposure.

## 1. Introduction

Feedstuffs represent the foundation of nutrition in animal husbandry, serving as the primary source of nutrients for farm animals and directly influencing their health, productivity, and the safety of animal-derived products [1]. Species belonging to the genera *Aspergillus*, *Fusarium*, and *Penicillium* frequently colonize cereals and other plant materials used in animal feed, both during the growing season and throughout harvesting, transport, and storage [2]. Environmental conditions favorable to fungal development—particularly elevated moisture and optimal temperatures—promote their proliferation and the synthesis of toxic secondary metabolites (mycotoxins), thereby significantly reducing the nutritional quality and safety of feedstuffs [3].

Globally, numerous monitoring studies have demonstrated that feed is often simultaneously contaminated with multiple mycotoxins, commonly 2–5 toxic compounds, generating cumulative or synergistic risks [4]. During feed manufacturing, the blending of multiple raw materials creates complex matrices that favor the co-occurrence of mycotoxins. Consequently, multi-mycotoxin contamination in livestock feed is now considered the rule rather than the exception. Recent large-scale surveys consistently report high prevalence rates worldwide. Studies showed that over 90% of livestock feed samples contained more than one mycotoxin, frequently involving aflatoxins, fumonisins, deoxynivalenol (DON), zearalenone (ZEA), and ochratoxin A (OTA). Similarly, a Kenyan survey reported multi-mycotoxin co-contamination in 96% of dairy and poultry feeds, while studies from Europe and South Asia documented co-occurrence rates ranging from 60% to nearly 100%, depending on feed type and region. Global monitoring programs further confirm that approximately 60–75% of finished feeds contain at least two mycotoxins, highlighting the importance of cumulative exposure assessment in feed safety evaluation [5].

In dairy cattle, even sub-threshold levels of these contaminants may impair health and productivity in the absence of overt exceedances of legal limits [6,7].

In Romania, climatic variability—characterized by alternating periods of drought and high humidity—combined with shortcomings in grain drying and storage practices, promotes contamination of feed materials with mycotoxigenic fungi [8]. At the European level, scenario analyses on climate-change impacts predict a potential expansion of the environmental range of toxigenic fungi and shifts in contamination profiles, particularly for AFB1 in southern and eastern regions of Europe [2,9]. Despite isolated investigations, there remains a pronounced need for applied, locally conducted studies that correlate fungal contamination, mycotoxin occurrence, and physiological effects in animals [10].

Mycotoxins act on the animal organism even at low concentrations, exerting hepatotoxic, nephrotoxic, immunosuppressive, estrogenic, or carcinogenic effects depending on the compound, exposure level, and individual susceptibility of the animal [11]. A relevant example is aflatoxin B1 (AFB1) produced by *Aspergillus flavus*, which, when ingested through feed, is metabolized in the liver to aflatoxin M1 (AFM1)—a compound excreted in milk and recognized for its carcinogenic potential [12]. Due to its thermal stability and ability to persist in processed milk, AFM1 represents a major public-health concern and is strictly regulated at the European level [13,14].

Recent studies from Mediterranean regions and other areas with high milk consumption have frequently detected AFM1 in raw and processed milk, sometimes at levels close to or exceeding regulatory limits, raising concerns about exposure among vulnerable groups such as infants and young children [15,16]. Other mycotoxins relevant to dairy cattle include OTA, DON, and ZEA, each with distinct toxic mechanisms and potential impacts on ruminant health [5,17,18].

The effects of mycotoxins do not always manifest acutely; in many cases, chronic exposure to subclinical doses may induce subtle metabolic and physiological alterations detectable only through laboratory analyses [19]. Assessing the metabolic status of animals through serum biochemical parameters therefore becomes a valuable tool for the early detection of imbalances induced by feed contamination. Frequently affected parameters include hepatic enzymes (ALP, AST, GGT), albumin, bilirubin, urea, creatinine, and lipid metabolites [20]. In experimental and field studies, exposure of dairy cows to naturally contaminated feed containing AFB1, DON, ZEA, and other mycotoxins has been associated with changes in these parameters, reduced voluntary feed intake, and decreased milk yield [21]. Recent reviews on ruminant production concludes that monitoring the biochemical blood profile (hepatic enzymes, nitrogen metabolites, lipids) is a key element in managing mycotoxin risk in dairy farms [22,23].

In this context, the present study addresses an issue of high relevance, with significant implications for animal nutrition, veterinary sciences, and food safety. The aim of this research was to evaluate the correlations between fungal contamination of feed, mycotoxin levels, and alterations in serum biochemical parameters in dairy cattle, as well as to investigate the presence of aflatoxin M1 in milk in order to assess the potential risk of transfer to consumers. By focusing on a specific production system in Romania and combining mycological analysis, multi-mycotoxin determination, and serum biochemical profiling, the study provides integrative data reflecting real farm conditions and contributes to the development of more effective management and monitoring strategies. These data may serve as a basis for risk assessments adapted to regional contexts, in line with “farm-to-fork” principles.

## 2. Results

### 2.1. Influence of Moisture on Fungal Load

Moisture values ranged from 9.97% in rapeseed meal to 61.40% in corn silage. Dry substrates (10–14%) predominantly supported *Aspergillus* spp., confirming its xerophilic behavior. In contrast, substrates with moisture levels above 50%—such as the mixed ration and corn silage—recorded substantial increases in *Fusarium* spp. and in total fungal counts, which reflected their susceptibility to secondary fermentation processes and microaeration.

**Figure 1:**
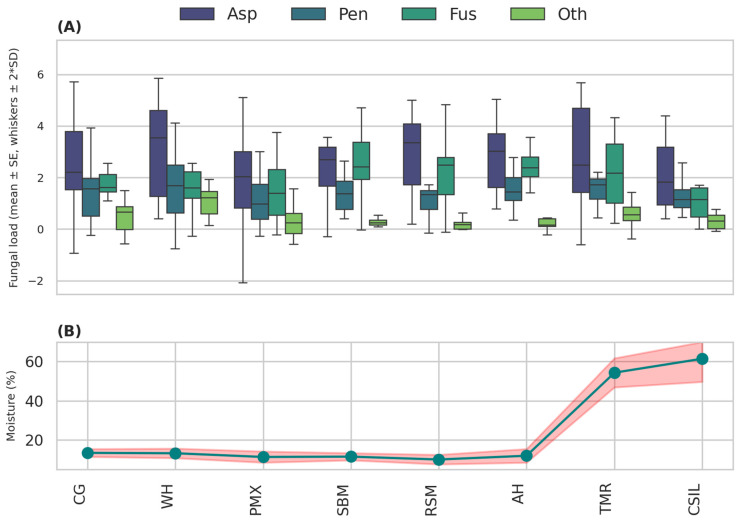
Normalized fungal load by sample type (**A**) and its relationship with moisture content (**B**). Central boxes represent mean ± SE, and whiskers indicate ±2 SD. *Asp*—*Aspergillus* spp.; *Pen*—*Penicillium* spp.; *Fus*—*Fusarium* spp.; *Oth*—other fungi. Feed types: corn (CG), wheat (WH), feed premix (PMX), soybean meal (SBM), rapeseed meal (RSM), total mixed ration (TMR), corn silage (CSIL), and alfalfa hay (AH).

These relationships between moisture and fungal genera are clearly represented in Figure 1, where fungal load escalation correlates with increased water content in the feed matrix.

The fungal assessment performed on feedstuffs intended for dairy cattle revealed a heterogeneous contamination profile, determined mainly by substrate composition and moisture content (Table 1). Total fungal counts ranged from 5.38 × 10^3^ CFU/g in rapeseed meal to 7.51 × 10^3^ CFU/g in corn silage, values that complies with the range of moderate fungal contamination typical of standard farm storage conditions. Mean values of approximately 6–7 × 10^3^ CFU/g indicate a fungal status that remained stable, although sufficiently active to support mycotoxin formation when favorable environmental conditions occur.

Figure 1 illustrates these patterns by integrating normalized fungal loads for each genus with the corresponding moisture levels of the samples. The plot confirms the strong association between moisture variability and fungal proliferation, particularly in high-moisture substrates such as total mixed ration and corn silage.

### 2.2. Distribution of Fungal Genera

*Aspergillus* spp. showed the highest mean levels in several feed categories, with maxima in the feed premix (3.32 × 10^3^ CFU/g), corn kernels (3.03 × 10^3^ CFU/g), and corn silage (2.92 × 10^3^ CFU/g). Its dominance in low-moisture substrates (10–14%) corresponds to storage-related contamination, as this genus adapts well to low water activity.

*Fusarium* spp. recorded its highest values in soybean meal (2.55 × 10^3^ CFU/g), the mixed ration (2.44 × 10^3^ CFU/g), and rapeseed meal (2.06 × 10^3^ CFU/g). These values indicate an origin linked to field contamination, because *Fusarium* species commonly occur on cereals and oilseed crops prior to harvest.

*Penicillium* spp. showed relatively consistent levels across all feed categories (1.06–1.73 × 10^3^ CFU/g), which confirmed its ecological potential in both dry and fermented substrates.

**Figure 2:**
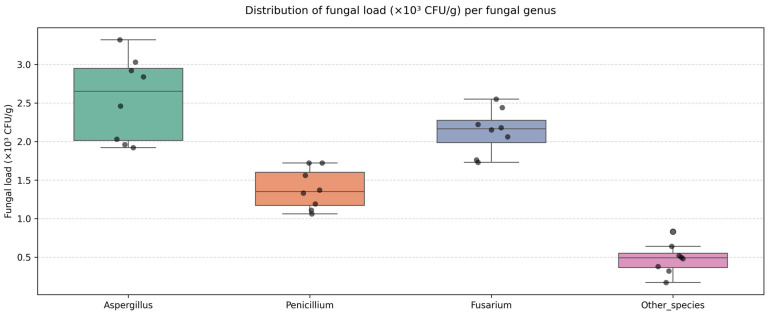
Distribution of fungal load (×10^3^ CFU/g) per fungal genus.

The boxplot in Figure 2 summarizes the distribution of fungal genera and supports the numerical trends: *Aspergillus* spp. displayed the highest median load, followed by *Fusarium* spp., whereas other fungal species remained low and relatively uniform.

### 2.3. Correlation Between Moisture Content (%) and Fungal Load (CFU/g) in Different Feed Matrices

The correlation analysis was performed between moisture content (%) and fungal load expressed as colony-forming units per gram (CFU/g) for each major fungal genus and total fungal load, revealing substantial variability among feed matrices.

Table 2 summarizes the significant relationships (*p* < 0.05) between moisture and each fungal group, highlighting clear differences between substrate categories.

In the case of *Aspergillus* spp., correlations with moisture ranged from moderately positive values in rapeseed meal (0.293 **), feed ration (0.173 **), and alfalfa hay (0.260 **), to negative values in wheat (−0.221 **) and feed premix (−0.191 **), as reflected in Table 2.

For *Penicillium* spp., most coefficients were negative in cereals and premix (e.g., −0.416 ** in feed premix), while slightly positive values appeared in fibrous feedstuffs such as alfalfa hay (0.145 *).

*Fusarium* spp. displayed the strongest positive correlations with moisture, particularly in wheat (0.558 **), feed premix (0.473 **), feed ration (0.162 *), and alfalfa hay (0.277 **), together with negative correlations in soybean meal (−0.525 **) and rapeseed meal (−0.362 **).

The other species category showed moderate positive correlations in whole or fibrous matrices, such as wheat (0.387 **) and feed premix (0.508 **), while negative values appeared in oilseed meals. Regarding total fungal load, positive correlations were observed in wheat (0.241 **), feed premix (0.159 *), feed ration (0.254 **), and alfalfa hay (0.398 **), whereas soybean meal recorded a negative correlation (−0.464 **).

In addition to moisture–fungus relationships, the interactions among fungal genera revealed further structural differentiation within the feed mycobiota.

The correlation patterns summarized in Table 3 reveal two dominant ecological mechanisms shaping the fungal communities across feed matrices: co-proliferation within compatible genera and antagonistic partitioning between genera with divergent ecological requirements.

First, the consistently strong positive correlations between *Aspergillus* spp. and total fungal load—present in all matrices, but particularly pronounced in feed ration (r = 0.688 **) and corn (r = 0.660 **)—suggest that *Aspergillus* spp. is an important representative of overall fungal biomass, especially under dry or moderately moist conditions. Likewise, *Fusarium* spp. showed uniformly strong associations with total fungi (0.543–0.760 **, depending on matrix), underscoring its ecological dominance in high-moisture substrates such as soybean meal and corn silage. These findings indicate that co-proliferation occurs primarily when environmental conditions simultaneously favor the physiology of a given genus and the broader fungal community.

Such negative correlations are consistent with competitive exclusion driven by ecological niche differentiation among filamentous fungi, a mechanism well documented in cereal- and feed-based matrices. The observed inverse relationships between *Aspergillus* spp. and *Fusarium* spp. in wheat (r = −0.228, *p* < 0.01) and rapeseed meal (r = −0.210, *p* < 0.01), as well as between *Penicillium* spp. and *Fusarium* spp. in feed premix (r = −0.382, *p* < 0.01), can be primarily explained by differences in water activity requirements. *Fusarium* spp. generally require higher moisture levels (aw ≥ 0.90–0.95) for growth and secondary metabolite production, whereas *Aspergillus* and *Penicillium* spp. are able to colonize substrates with substantially lower aw values, giving them a competitive advantage in dry or processed feed materials [24].

In addition to moisture constraints, oxygen availability plays a role in shaping fungal community structure. *Fusarium* spp. are strictly aerobic and less competitive under microaerobic conditions typical of compacted or finely milled feed premixes, while *Aspergillus* and *Penicillium* spp. exhibit greater metabolic flexibility and tolerance to reduced oxygen diffusion [25]. This mechanism is consistent with the stronger negative correlation observed in feed premix, where processing intensity and low porosity limit oxygen availability.

Differences in pH tolerance and nutrient utilization strategies further contribute to the observed antagonism. *Aspergillus* and *Penicillium* spp. tolerate broader pH ranges and possess versatile enzymatic systems that allow efficient exploitation of residual carbohydrates, lipids, and proteins in processed matrices, whereas *Fusarium* spp. are more competitive in intact plant tissues and less altered substrates [26]. Together, these physiological and metabolic differences explain the consistent negative correlations observed between these genera and support the interpretation that substrate properties and processing degree are key drivers of fungal community shifts in compound feeds.

**Figure 3:**
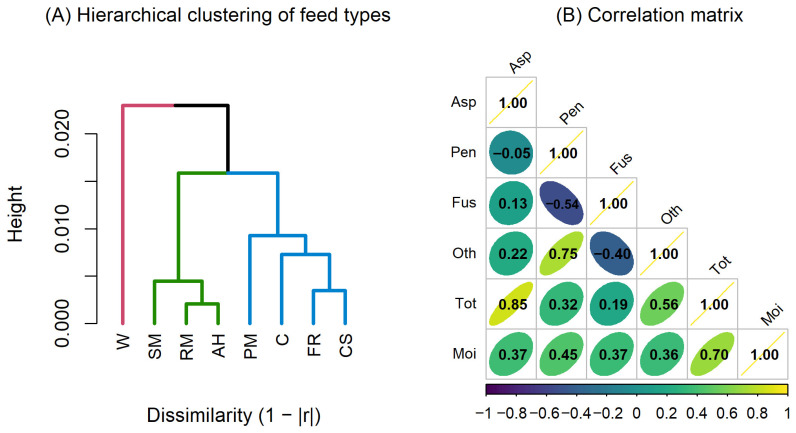
(**A**) Correlation matrix (*Asp* = *Aspergillus*, *Pen* = *Penicillium*, *Fus* = *Fusarium*, *Oth* = other fungi, *Tot* = total fungi, *Moi* = moisture) showing moisture-driven differentiation of fungal groups. (**B**) Hierarchical clustering of feed types (*C* = Corn, *W* = Wheat, *PM* = Feed Premix, *SM* = Soybean meal, *RM* = Rapeseed meal, *FR* = Feed ration, *CS* = Corn silage, *AH* = Alfalfa hay) reflecting the separation between low-moisture, storage-associated substrates and high-moisture, *Fusarium*-rich matrices.

Overall, the combined information in Figure 3 outlines a differentiated correlation pattern between moisture and fungal development, strongly dependent on feed type, together with clear profiles of co-occurrence or antagonism among dominant fungal genera. Moisture-rich substrates favored hydrophilic and plant-associated taxa such as *Fusarium* spp., while dry substrates promoted competition among xerophilic storage fungi.

The correlation matrix (Figure 3A) shows that moisture strongly promotes *Fusarium* spp. development in high-moisture substrates, while dry cereals are dominated by *Aspergillus* spp. and *Penicillium* spp., which correlate negatively or weakly with moisture. These patterns are reflected in the hierarchical clustering of feed types (Figure 3B), where corn, wheat, and premix form a low-moisture cluster characterized by storage fungi, oilseed meals occupy an intermediate position, and the feed ration and corn silage cluster together as high-moisture matrices dominated by hygrophilic fungal communities. Together, Figure 3A,B confirm moisture as the primary factor structuring fungal profiles across feedstuffs.

### 2.4. Monthly Dynamics of Mycotoxin Contamination in Feed Matrices

**Figure 4:**
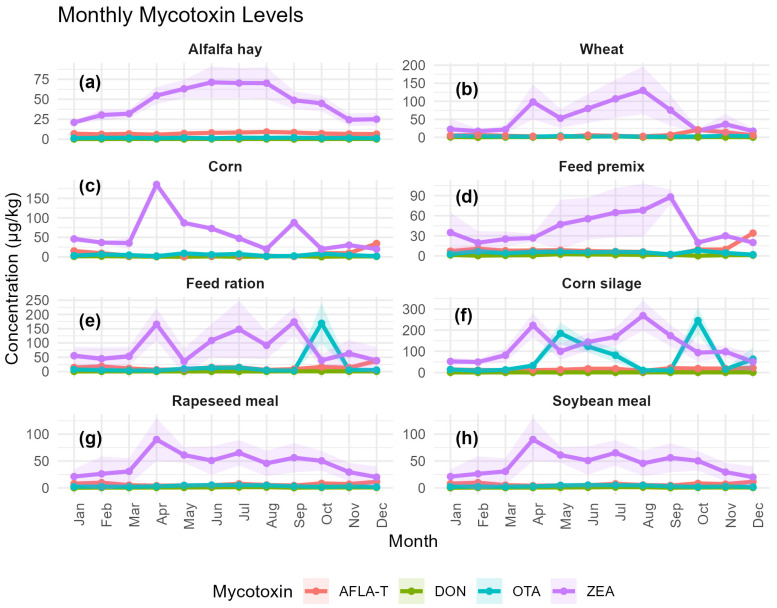
Monthly dynamics of mycotoxin concentrations across eight feed matrices (**a**) alfalfa hay, (**b**) wheat, (**c**) corn, (**d**) feed premix, (**e**) feed ration, (**f**) corn silage, (**g**) rapeseed meal, and (**h**) soybean meal. Monitored mycotoxins (AFLA-T, DON, OTA, ZEA). Values are expressed in µg/kg (AFLA-T, OTA, ZEA) and mg/kg (DON).

The monthly monitoring of AFLA-T, OTA, ZEA, and DON across the eight-feed ma-trices revealed clear temporal dynamics strongly dependent on feed type and moisture content, as illustrated in Figure 4a–h and supported by the dataset generated by this study.

The highest ZEA concentrations in the entire dataset occurred in the high-moisture substrates. Corn silage (Figure 4f) showed two exceptional peaks, first 222.59 ± 19.12 µg/kg in April and subsequently the annual maximum of 268.64 ± 23.57 µg/kg in August, followed by a secondary rise in October. The mixed feed ration (Figure 4e) displayed an analogous seasonal dynamic, culminating in a September maximum of 173.95 ± 16.82 µg/kg. By contrast, soybean meal and rapeseed meal (Figure 4g,h) remained within moderate ranges, typically 20–90 µg/kg, and showed weaker seasonal signals. Alfalfa hay (Figure 4a) exhibited a predictable early-summer elevation, with values rising from 20.96 ± 0.70 µg/kg in January to 77.60 ± 3.20 µg/kg in June, before gradually decreasing toward winter.

In comparison with ZEA, total aflatoxins (AFLA-T) remained substantially lower across all feed types, with most monthly means below 10–15 µg/kg. Nonetheless, a clear storage-related seasonal pattern was evident. In corn (Figure 4c), AFLA-T decreased from 15.06 ± 0.61 µg/kg in January to a minimum range of 1.00 ± 0.06 to 3.10 ± 0.07 µg/kg between March and September, followed by a pronounced rise to 34.07 ± 0.57 µg/kg in December. A similar late-season increase was evident in feed premix (34.07 ± 0.57 µg/kg in December) and feed ration (37.86 ± 4.89 µg/kg in December), consistent with aflatoxin accumulation during extended storage. Oilseed meals remained comparatively stable, with soybean meal showing values between 4.16 ± 0.63 and 11.54 ± 0.92 µg/kg, while rapeseed meal displayed similar low-amplitude fluctuations. Alfalfa hay maintained uniformly low AFLA-T levels throughout the year (approximately 6–9 µg/kg).

Among the four monitored toxins, deoxynivalenol (DON) exhibited the most stable profile. In corn (Figure 4c), DON fluctuated only modestly between 0.20 ± 0.07 and 1.74 ± 0.01 mg/kg. Wheat (Figure 4b) showed a moderate summer increase, reaching 2.52 ± 0.27 mg/kg in July, but otherwise remained stable. DON concentrations in feed premix ranged from 0.91 ± 0.49 to 2.61 ± 0.63 mg/kg, while soybean meal and rapeseed meal displayed annual means between 0.36–1.61 mg/kg, without marked seasonality. DON remained uniformly low in corn silage (0.47–0.83 mg/kg) and alfalfa hay (0.21–0.32 mg/kg), further confirming its low temporal variability compared with the other mycotoxins.

Total aflatoxins (AFLA-T) remained considerably lower than ZEA or OTA, with typical monthly means below 10–15 µg/kg. Corn exhibited a classical storage-related accumulation pattern: from 15.06 ± 0.61 µg/kg in January, levels dropped to 1.00 ± 0.06–3.10 ± 0.07 µg/kg between March and September, followed by a sharp increase to 34.07 ± 0.57 µg/kg in December. Feed premix and feed ration mirrored this late-season increase, reaching 34.07 ± 0.57 µg/kg (December) and 37.86 ± 4.89 µg/kg (December), respectively. Soybean and rapeseed meals remained within narrow ranges (mostly 4–12 µg/kg).

**Figure 5:**
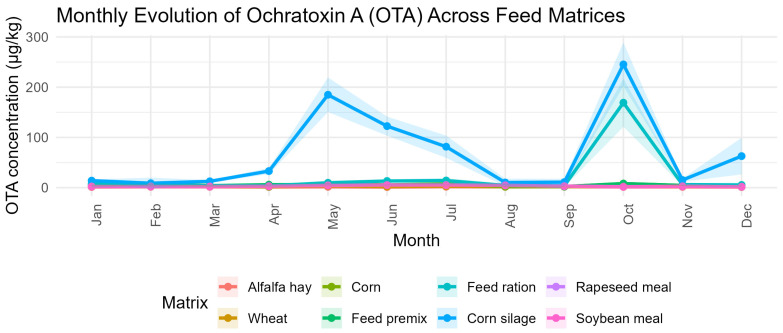
Monthly dynamics of Ochratoxin A (OTA) (µg/kg) across eight feed matrices.

Across most feed matrices, OTA remained consistently low, with narrow monthly variability reflected by small standard deviations (Figure 5). In corn, OTA concentrations ranged from 1.85 ± 0.09 µg/kg (August) to 9.07 ± 0.09 µg/kg (May). In wheat, values fluctuated between 1.90 ± 0.45 µg/kg (April) and 4.51 ± 0.77 µg/kg (January). Feed premix showed a similar low-level profile, with OTA spanning from 2.30 ± 0.06 µg/kg (September) to 8.18 ± 0.16 µg/kg (October).

Oilseed meals also maintained low OTA levels: soybean meal ranged between 1.23 ± 0.65 µg/kg (December) and 5.29 ± 1.39 µg/kg (June), while rapeseed meal varied from 1.90 ± 0.56 µg/kg (December) to 5.29 ± 1.39 µg/kg (June) with minimal month-to-month amplitude. Alfalfa hay remained uniformly low as well, ranging from 1.23 ± 0.65 µg/kg (December) to 1.93 ± 0.80 µg/kg (September).

In contrast, the most pronounced OTA events were observed in corn silage, which displayed two extreme peaks: 184.86 ± 17.19 µg/kg in May and 245.22 ± 21.74 µg/kg in October. Similarly, feed ration exhibited a dramatic contamination episode in October, reaching 169.09 ± 23.91 µg/kg, compared with background levels typically between 3.76 ± 1.19 µg/kg (April) and 14.26 ± 2.37 µg/kg (July).

The Kruskal–Wallis analysis confirmed that the four mycotoxins exhibited statistically distinct concentration profiles within every feed matrix (all *p* < 0.001), indicating that contamination patterns are mycotoxin-specific rather than uniform across substrates.

In alfalfa hay and wheat, the strongest differentiation was driven by consistently higher ZEA levels compared with OTA, AFLA-T, and DON. Corn showed a similar pattern, with ZEA dominating and AFLA-T contributing only a late-season increase. Processed ingredients such as feed premix and oilseed meals also displayed significant separation among mycotoxins, demonstrating that processing does not homogenize contamination and that ZEA remains predominant across plant-derived substrates. High-moisture matrices (feed ration and corn silage) showed distinct mycotoxin profiles characterized by intense ZEA peaks and episodic OTA spikes, while AFLA-T and DON remained comparatively low. Overall, these results statistically support the trends observed in the monthly plots: ZEA is consistently the most abundant toxin, OTA shows intermittent high elevations, AFLA-T increases mainly late in the year, and DON remains the least variable contaminant across all feed matrices.

### 2.5. Relationship Between Aflatoxin Contamination in Feed, AFM1 Transfer into Milk, and Biochemical Alterations in Dairy Cattle

The integrated analysis of aflatoxin contamination in feed and the corresponding levels of AFM1 detected in raw milk indicates a direct mechanistic relationship between dietary exposure and hepatic biotransformation in dairy cattle. Aflatoxin B1 (AFLA-B1), the predominant component of total aflatoxins (AFLA-T) in feedstuffs, undergoes phase I biotransformation in the liver through 4-hydroxylation mediated by cytochrome P450 enzymes, generating AFM1, which is subsequently excreted into milk. Reported conversion rates range between 0.3% and 6% of the ingested AFB1, sufficiently high for fluctuations in AFLA-T concentrations in feed to be reflected in milk AFM1 levels [27,28].

In the present study, the seasonal increases of AFLA-T observed in corn, corn silage, and mixed ration during late storage (e.g., December peaks of 34 µg/kg in corn, 37.86 ± 4.89 µg/kg in feed ration, and 21.86 ± 1.60 µg/kg in corn silage) are consistent with the classical pattern of AFM1 enrichment in milk during periods of elevated exposure.

This relationship is not only toxicological but also metabolic. Elevated AFM1 in milk serves as a biomarker of chronic hepatic exposure, and in many reports correlates with measurable biochemical alterations. Aflatoxins exert their hepatotoxicity through oxidative stress, mitochondrial dysfunction, disruption of hepatocyte membrane integrity, and interference with the urea cycle [29].

These mechanisms lead to increases in hepatic injury markers (ALT, AST), cholestasis-associated enzymes (ALP, GGT), and total bilirubin, alongside decreases in hepatic synthesis markers such as albumin, total proteins, cholesterol, and triglycerides. In advanced or prolonged exposure, creatinine may rise due to secondary renal involvement, while serum urea may fall as a result of impaired hepatic conversion of ammonia to urea. These metabolic pathways have been widely described in experimental and field studies on aflatoxin exposure in dairy cattle and small ruminants [30].

**Figure 6:**
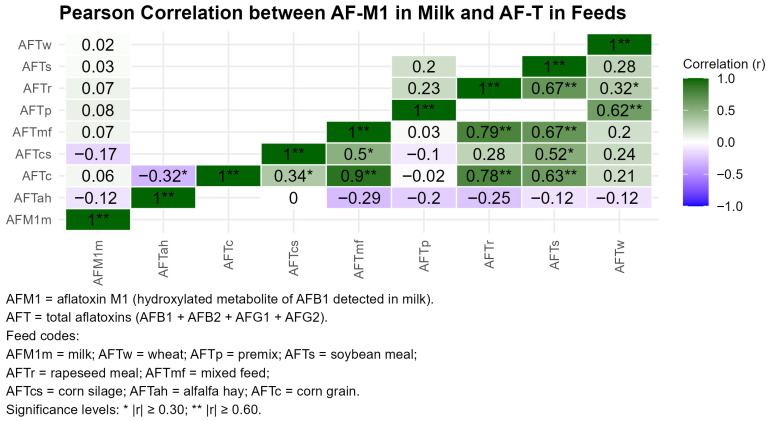
Pearson correlations between AFM1 in milk and AFLA-T across feed matrices.

The heatmap illustrates (Figure 6) the pairwise Pearson correlations between AFM1 concentrations in raw milk and AFLA-T levels across all eight feed matrices. Strongest positive associations were observed for corn (r = 1.00 **, *p* < 0.01), corn silage (r = 0.79 **, *p* < 0.01), and mixed ration (r = 0.67 **, *p* < 0.01), consistent with their higher AFLA-T levels during late-season storage. Negative or negligible correlations were detected for low-contamination matrices such as wheat, soybean meal, and alfalfa hay. The plot highlights the central role of high-moisture and bulk feed components (corn, silage, ration) in predicting AFM1 transfer to milk.

### 2.6. Transfer of Aflatoxins from Feed to Milk and Variation of Serum Biochemical Parameters

The monthly monitoring of total aflatoxins (AFLA-T) across the eight feed matrices revealed pronounced seasonal and substrate-specific variability, with clear distinctions between dry cereals, oilseed meals, mixed rations, and high-moisture forages. According to the dataset used in this study, annual mean AFLA-T levels were 7.71 ± 9.08 µg/kg in corn, 6.64 ± 5.35 µg/kg in wheat, approximately 6–8 µg/kg in soybean and rapeseed meals, 7.71 ± 9.08 µg/kg in feed premix, 14.18 ± 8.64 µg/kg in the mixed feed ration, and 15.39 ± 4.51 µg/kg in corn silage. The most substantial temporal fluctuations were observed in moisture-rich substrates: in the mixed ration, AFLA-T varied between 5.86 ± 1.29 µg/kg in August and 37.86 ± 4.89 µg/kg in December, while corn silage ranged from 11.25 ± 1.33 µg/kg in March to 21.86 ± 1.60 µg/kg in December. Corn kernels displayed the clearest storage-driven trajectory, decreasing steadily from 15.06 ± 0.61 µg/kg in January to values as low as 1.00 ± 0.06–2.23 ± 0.14 µg/kg between April and September, followed by a sharp resurgence to 34.07 ± 0.57 µg/kg at the end of the year. These trends confirm that substrate moisture, storage duration, and environmental conditions collectively contribute to the seasonal intensification of AFLA-T contamination.

Despite these marked fluctuations in feed exposure, AFM1 concentrations in milk remained consistently low throughout the monitoring period. All monthly values were far below the EU maximum limit of 0.05 µg/kg for raw milk, ranging from 0.0020 ± 0.0000 µg/kg during April–August to a peak of 0.0093 ± 0.0104 µg/kg in March. Importantly, AFM1 did not increase in parallel with the late-autumn and winter rise in AFLA-T observed across several feed matrices. While corn, mixed ration, and silage displayed maximum AFLA-T loads in December, the corresponding AFM1 value remained moderate at 0.0038 ± 0.0016 µg/kg. This decoupling reflects a partially buffered, non-linear carry-over mechanism influenced by hepatic biotransformation, ruminal degradation, milk yield, and individual physiological variation, consistent with reported conversion efficiencies of 0.3–6% of ingested AFB1.

Although correlations were initially explored between total aflatoxins (AFLA-T) in feed and AFM1 in milk, AFM1 formation is biologically linked specifically to dietary aflatoxin B1 (AFB1). Therefore, correlations involving AFLA-T were interpreted as indirect associations, reflecting the contribution of AFB1 within the total aflatoxin pool.

Across the twelve-monthly observations, Pearson coefficients linking AFM1 to AFLA-T from individual feed ingredients were uniformly weak and statistically non-significant, typically ranging between −0.15 and +0.26. Even the cumulative AFLA-T intake derived from all matrices combined exhibited only a negligible association with AFM1 (r ≈ −0.09, *p* > 0.7). These findings demonstrate that milk AFM1 levels are shaped by a complex interplay of dietary, physiological, and metabolic factors rather than by contamination in any single feed component.

The serum biochemical profile of the cows remained within physiological limits but exhibited coherent seasonal trends that paralleled, to some extent, fluctuations in dietary mycotoxin exposure. Albumin showed an annual mean of 32.41 ± 4.86 g/L, with a nadir of 26.73 ± 4.96 g/L in February and a maximum of 34.87 ± 3.20 g/L in March. Total serum protein remained stable at approximately 74–75 g/L across the year. Cholesterol varied between 175.13 ± 37.86 mg/dL in August and 216.53 ± 60.97 mg/dL in April, while triglycerides reached their lowest values during midsummer, decreasing to 18.27 ± 4.32 mg/dL in July and 16.67 ± 6.31 mg/dL in August, patterns consistent with seasonally elevated metabolic demands.

Markers of hepatic function also remained within normal physiological intervals, though subtle seasonal modulations were apparent. Alkaline phosphatase (ALP) averaged 102.14 ± 42.37 U/L but increased markedly toward the end of the year, reaching 156.87 ± 62.76 U/L in December. Transaminases showed milder variations: ALT fluctuated between 22.40 ± 4.94 U/L in January and 29.13 ± 6.52 U/L in December, whereas AST ranged from 78.13 ± 15.67 U/L in August to 116.07 ± 30.41 U/L in January. GGT varied between 22.20 ± 8.69 U/L in October and 40.00 ± 17.37 U/L in September. Total bilirubin remained low for most of the year (0.05–0.29 mg/dL), with a single pronounced increase in October, reaching 1.05 ± 0.43 mg/dL. These patterns are compatible with mild, subclinical metabolic adjustments rather than overt hepatotoxicity.

Renal and metabolic indices also followed consistent seasonal trajectories. Serum urea displayed one of the broadest ranges, increasing from 8.87 ± 5.53 mg/dL in July to a maximum of 35.80 ± 6.61 mg/dL in March, while creatinine remained stable within physiological limits (overall 0.71 ± 0.18 mg/dL), with slight elevations in months characterized by higher AFM1 levels. Serum calcium concentrations, ranging from 7.48 ± 0.94 to 10.18 ± 1.15 mg/dL, remained tightly regulated throughout the year, indicating preserved mineral balance.

### 2.7. Multivariate Assessment of Aflatoxin Exposure and Implications on Serum Biochemical Profiles

Hepatic status was derived from a panel of routine biochemical markers reflecting hepatocellular integrity and biliary function (ALT, AST, GGT, ALP, total bilirubin, and total protein). Each parameter was classified as low, within reference range, or high according to standard physiological intervals. For each animal, the number of out-of-range values was mediated into a composite hepatic load score. Individuals without deviations (score = 0) were assigned to the Normal group, whereas those exhibiting one or two mild abnormalities (score = 1–2) were classified as Borderline. Cases with ≥3 abnormalities were rare and therefore excluded from discriminant analysis [31].

Consequently, the hepatic status reflects the global biochemical profile rather than isolated parameter fluctuations, enabling a more robust evaluation of subtle functional alterations.

After complete-case filtering for linear discriminant analysis, the dataset contained a highly unbalanced structure (92% Normal, 8% Borderline). This explains the higher density of LD1 values around the Normal cluster. The LD1 discriminant function was predominantly driven by the multivariate biochemical structure (PC1 and PC3), while AFLA_T and AFLA_M1 contributed minimally. As a result, Normal group projected positive LD1 values, whereas Borderline groups tended to shift toward lower LD1 values, with partial overlap consistent with the mild, subclinical nature of the deviations defining the Borderline group.

The multivariate evaluation of aflatoxin exposure demonstrated that, despite the generally weak linear associations observed at the univariate level, a coherent biochemical signal emerged when the serum profile was examined in a global, multivariate framework. As summarized in Table 4, simple Pearson and Spearman correlations between AFLA-T in feed and individual biochemical parameters were negligible (r values between −0.15 and +0.26), confirming that isolated serum markers are insufficient to capture the physiological burden of low-level aflatoxin exposure.

However, multivariate approaches revealed structured patterns. PERMANOVA showed that AFLA-T categories did not significantly explain biochemical variation (F = 1.73, R^2^ = 0.033, *p* = 0.115), whereas AFLA-M1 categories explained more than 7% of the total multivariate variance (F = 8.01, R^2^ = 0.073, *p* = 0.002). This indicates that internal exposure, rather than feed contamination itself, aligns more closely with physiological responses. MANOVA applied to the first three principal components of the biochemical dataset confirmed this pattern: the effect of AFLA-M1 categories was robust (Pillai = 0.198, F = 8.24, *p* = 5.9 × 10^−5^), while AFLA-T categories showed only marginal association (*p* = 0.068). Moreover, canonical correlation analysis demonstrated a highly significant latent correlation structure (*p* < 10^−13^), reinforcing the idea that low-level AFM1 accumulation is biologically meaningful even in the absence of overt clinical abnormalities.

To further explore whether biochemical alterations cluster into physiologically relevant groups, we constructed a composite Hepatic Status classification based on deviations in six hepatic markers (ALT, AST, GGT, ALP, total bilirubin, total protein). As detailed in the Methods and supported by Table 4, animals without biochemical deviations were assigned to the *Normal* group, whereas those showing one or two mild abnormalities—consistent with subtle, subclinical hepatic adaptations—were classified as *Borderline*. Because cases with ≥3 abnormalities were rare, discriminant analyses focused on these two groups.

**Figure 7:**
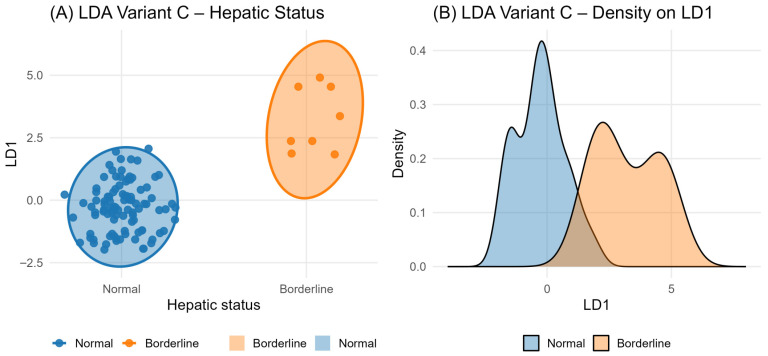
Linear discriminant projection of hepatic status based on AFM1 exposure and multivariate biochemical profile. (**A**): The LDA projection; (**B**) The LD1 density plot divergence between Normal and Borderline groups.

The LDA model integrating aflatoxin exposure (AFLA-T, AFLA-M1), biochemical principal components (PC1–PC3), and organ-level loads produced a single linear discriminant (LD1), whose behavior is illustrated in Figure 7A. The 95% confidence ellipses demonstrate that animals in the *Borderline* category are consistently projected toward lower LD1 values, while *Normal* animals cluster around positive or near-zero LD1 values. This shift reflects underlying multivariate biochemical differences driven primarily by PC1 and PC3—components dominated by liver-associated enzymes—rather than by aflatoxin levels, whose discriminant coefficients were minimal. The class distribution was highly unbalanced (92% Normal vs. 8% Borderline), which also explains the denser LD1 cluster around the Normal centroid.

The LD1 density curves in Figure 7B further highlight this pattern: the *Borderline* group exhibits a shift of the density mass toward negative LD1 values, indicating a coherent biochemical deviation, yet the substantial overlap with the *Normal* distribution emphasizes that these effects remain subtle and subclinical. This behavior is physiologically consistent with chronic low-dose aflatoxin exposure: AFM1 accumulates at microgram-per-liter levels, sufficiently sensitive to reflect metabolic adjustment, but not intense enough to produce distinct or clinically abnormal biochemical signatures.

Taken together, the combined evidence from PERMANOVA, MANOVA, CCA, and LDA (summarized in Table 4) supports the conclusion that AFM1 is a more sensitive indicator of physiological impact than AFLA-T, even at concentrations far below regulatory thresholds. Internal exposure correlates with structured, multivariate biochemical changes, while feed contamination levels alone do not capture the metabolic response. The LDA visualizations provide an intuitive representation of these findings, demonstrating that animals classified as Borderline occupy a consistent but overlapping region of the biochemical space defined by LD1, reflecting mild, coordinated biochemical deviations rather than overt hepatic injury.

## 3. Discussion

### 3.1. Fungal Ecology and Determinants of Community Structure in Feed Matrices

The results of this study clearly demonstrate that moisture is the primary ecological determinant shaping fungal community composition in livestock feed, functioning both as a regulator of metabolic activity and as a selective environmental filter for different fungal genera. This differentiated distribution is fully consistent with established ecological mechanisms governing fungal colonization, survival, and competitive interactions.

Inverse relationship between *Aspergillus* spp. and moisture content, confirming the strongly xerophilic nature of this genus was important to be notices. *Aspergillus* is widely recognized as an organism capable of germinating and producing secondary metabolites under very low water activity (aw < 0.75), allowing it to dominate dry substrates such as stored cereals and premixes [25]. In more heterogeneous matrices such as alfalfa—where micro-niches of variable humidity are present—*Aspergillus* may coexist with more hydrophilic species.

Fhe strong positive correlations between *Fusarium* spp. and moisture reflect its hydrophilic and phytopathogenic character. *Fusarium* proliferates rapidly under conditions of elevated water activity (a_w_ > 0.90), both in field crops and in ensiled materials, where moisture remains high. Numerous studies have shown that *Fusarium* spp. colonization intensifies in humid substrates, leading to increased production of DON and ZEA [32]. The correlations identified in wheat, premix, and alfalfa in our dataset are therefore fully aligned with known biological principles, confirming that moisture accumulation—whether due to field conditions or post-harvest absorption—is a strong predictor of mycotoxin risk.

*Penicillium* spp., which exhibits an intermediate ecological profile, showed positive correlations in fiber-rich feeds but negative associations in processed or low-moisture substrates such as premixes and certain cereals. This pattern supports the hypothesis that Penicillium thrives under conditions of moderate moisture, micro-aeration, and intermediate temperature, which are known to favor the development of ochratoxin A in inadequately stored feeds [33].

Beyond moisture effects, the relationships between fungal genera indicate clear patterns of co-colonization and fungal antagonism. Strong positive correlations between *Fusarium* spp. and total fungal counts across multiple matrices suggest that *Fusarium* spp. often becomes a dominant component of field and storage ecosystems, creating complex fungal assemblages that include both pathogenic and saprophytic species. In contrast, the consistent negative correlations between *Aspergillus* spp. and other genera may reflect the production of antifungal secondary metabolites, a phenomenon well documented in competitive storage environments [34].

Overall, these results demonstrates that fungal communities in feed are structured by moisture availability, substrate characteristics, and fungal interactions, with each genus occupying a distinct ecological niche. This ecological partitioning has direct implications for predicting mycotoxin profiles in different feed matrices and highlights the need for moisture-focused prevention strategies in post-harvest and storage management [35].

Overall, these results demonstrate that fungal communities in feed are structured by moisture availability, substrate characteristics, and interspecific interactions, with each genus occupying a distinct ecological niche. It should be acknowledged that fungal identification was restricted to the genus level, which precludes species-specific attribution of toxigenic potential. Nevertheless, the genus-level approach was intentionally adopted to capture broad ecological patterns relevant to feed hygiene and community structure, rather than to perform species-level toxigenic profiling.

### 3.2. Monthly Dynamics of Mycotoxins in Feed: Chemical Stability, Ecological Drivers, and Toxicological Implications

The monthly evolution of AFLA-T, OTA, ZEA and DON across the different feed matrices used in dairy cattle nutrition revealed a markedly non-linear behavior, characterized by sharp increases, transient reductions, and periods of re-accumulation. This pattern reflects not the direct dynamics of fungal proliferation, but rather the contrasting chemical stabilities of each mycotoxin and the ecological instability of high-moisture substrates such as corn silage and feed ration (FR). In such matrices, fluctuations in water activity, oxygen gradients, compaction heterogeneity, and fermentation processes create microenvironments that stimulate or suppress secondary metabolite formation independently of fungal biomass. These mechanisms are well documented in ecological and food-chain mycotoxin studies and demonstrate the importance of understanding toxin persistence beyond fungal growth alone [36,37].

Across the study year, ZEA and OTA exhibited the largest monthly amplitudes, with variations exceeding 10- to 20-fold in corn silage and TMR. For example, OTA increased from approximately 12 µg/kg in early spring to more than 240 µg/kg in autumn, a pattern consistent with its exceptional stability under anaerobic or semi-anaerobic conditions and its capacity to accumulate even when fungal activity is low. ZEA showed a similarly irregular pattern, with a pronounced rise during late winter–spring (40 → 160 µg/kg), a partial decline during summer, and a re-accumulation in early autumn. These cyclical trends align with studies indicating that zearalenone biosynthesis is triggered by alternating nutritional stress and shifts in oxidative balance rather than by continuous fungal growth [38].

In contrast, DON displayed the most predictable behavior, remaining within relatively narrow intervals (0.2–1.6 mg/kg). This stability is consistent with the well-known chemical resilience of trichothecenes, which resist degradation under both aerobic and anaerobic conditions and exhibit minimal seasonal fluctuation once incorporated into the feed matrix [39]. AFLA-T, while less variable than OTA or ZEA, showed a characteristic storage-related trend: moderate values during early and mid-year, followed by a decline to minimal levels in midsummer (1–2 µg/kg), and a pronounced resurgence in late-autumn and winter (up to 34 µg/kg). This profile reflects the interplay between declining oxygen exposure in stored grain, shifts in temperature gradients, and the persistence of aflatoxins even after fungal activity slows—a phenomenon repeatedly observed in post-harvest systems [34].

These findings indicate that mycotoxin production reflects environmental and physiological stress responses rather than fungal biomass, which limits the reliability of fungal counts for toxicological risk assessment. In corn silage, fungal load remained relatively constant around 10^3^–10^4^ CFU/g, yet OTA displayed a twenty-fold increase toward autumn. Similarly, declines in *Aspergillus* spp. abundance during summer coincided with subsequent increases in AFLA-T during winter. Such decoupling between fungal populations and toxin concentrations supports the concept that mycotoxins are ecological stress metabolites rather than proxies for fungal biomass. Environmental triggers—including freeze–thaw cycles, oxygen limitation, nutrient depletion, and interspecies competition within the silage microbiome—activate biosynthetic gene clusters responsible for secondary metabolism without requiring fungal proliferation [35].

From a toxicological perspective, these findings have direct implications for risk management. OTA and ZEA emerged as the most climatically sensitive toxins, responding strongly to microenvironmental instability within the silage mass; therefore, their monitoring should be performed monthly, particularly during spring and autumn transitions. AFLA-T requires intensified control during cold-season storage, when increases were most pronounced. By contrast, DON can be monitored seasonally due to its relatively stable behavior. Most importantly, corn silage and TMR represent the matrices of greatest vulnerability because their structural and physicochemical characteristics support the formation, persistence, and re-accumulation of multiple mycotoxins simultaneously. This aligns with recent evidence highlighting mixed-mycotoxin exposure as the rule rather than the exception in ruminant feeding systems [40,41].

### 3.3. Implications of AFM1 Formation and Aflatoxin Contamination Patterns in Feed

The finding that AFM1 concentrations in milk remained consistently below 0.01 µg/kg throughout the year, despite marked seasonal fluctuations in total aflatoxins (AFLA-T) across several feed matrices, is fully consistent with the established toxicokinetic literature on aflatoxin carry-over in dairy cattle. It should be noted that AFM1 formation is mechanistically linked exclusively to dietary aflatoxin B1 (AFB1); therefore, associations involving total aflatoxins (AFLA-T) should be interpreted only as indirect indicators reflecting the contribution of AFB1 within the total aflatoxin pool. Accordingly, the absence of a strong linear relationship between AFLA-T in feed and AFM1 in milk is expected under real farm conditions.

Analysis indicate that only a small fraction of ingested AFB1 is hydroxylated to AFM1 by hepatic CYP1A2 and CYP3A4, with transfer rates typically ranging between 0.3–6% depending on dose, metabolic status, and milk yield [42]. Controlled challenge studies further demonstrate that, at moderate intake levels, dairy cows can maintain stable hepatic function without marked elevations in standard serum biomarkers, even when AFM1 becomes detectable in milk [43]. This pattern is reflected in the present dataset: despite AFM1 fluctuations between 0.0020 and 0.0093 µg/kg, hepatocellular enzymes (ALT, AST, GGT) exhibited only mild seasonal shifts, and decreases in albumin or total protein were small and clinically irrelevant, suggesting metabolic adaptation rather than overt cytotoxicity.

The lack of a clear linear correlation between AFLA-T in feed and AFM1 in milk is expected, given that real ingestion, individual metabolic conversion rates, and dilution through milk yield heavily modulate the dose–response relationship. Recent reviews indicate that AFB1 → AFM1 conversion can vary 10-fold between herds exposed to comparable dietary concentrations, and that AFM1 equilibrium in milk may take several days to stabilize following changes in dietary AFB1 [44]. In this context, the weak correlations observed here (r ≈ −0.15 to +0.26 for individual feed matrices and r ≈ −0.09 for cumulative AFLA-T) likely reflect a combination of unmeasured behavioral and physiological variables such as feed intake variability, metabolic efficiency, and heterogeneity between feed batches.

From a clinical perspective, the absence of strong associations between AFM1 and classical liver injury markers is relevant, and indicates that the exposure levels encountered here fall within a “subclinical risk zone”—a condition well documented in adult ruminants, which exhibit greater resilience to aflatoxins compared to monogastric animals [43]. Nonetheless, the presence of moderate negative correlations with albumin and total protein and subtle positive tendencies in creatinine and urea may signal low-grade metabolic strain compatible with chronic low-intensity exposure. Similar subtle biochemical modulations have been reported in chronic AFB1/AFM1 studies, where gross elevations in ALT/AST are absent, but fine adjustments in protein and nitrogen metabolism emerge [45].

An important contextual factor is that cattle in this study were simultaneously exposed to other mycotoxins—ZEA, DON, OTA—documented in the same feed matrices. Global surveillance consistently shows that co-occurring mycotoxins are the rule, not the exception, and that their effects may be additive or synergistic even when each compound individually is below regulatory limits [46]. In this context, the weak linear relationships between AFM1 and serum biochemistry do not preclude combined toxicodynamic interactions involving estrogenic, oxidative, or immunomodulatory mechanisms of ZEA, DON, and OTA. Indeed, such complex interactions are often more effectively captured through multivariate statistical approaches, which the present study also applied (PERMANOVA, MANOVA, CCA, LDA).

Overall, the results support the conclusion that under real farm conditions, AFLA-T contamination in feed leads to limited and buffered AFM1 transfer, consistently below regulatory thresholds and without clinically relevant hepatotoxicity. However, the coexistence of multiple mycotoxins, the ecological variability in contaminated substrates, and the subtle shifts in serum markers justify the implementation of integrated monitoring programs that combine feed analysis, milk residues, and biochemical profiling. Multivariate statistical tools—PCA, PERMANOVA, CCA, LDA—provide a more robust understanding of both direct and indirect effects and offer an improved assessment of physiological impact under chronic low-dose exposure scenarios.

### 3.4. Aflatoxin Exposure, AFM1 Carry-Over, and Multivariate Biochemical Adaptation Under Low-Dose Chronic Conditions

Overall, the results of this study reveal a biologically coherent and literature-consistent pattern linking feed contamination, AFM1 transfer, and subtle multivariate biochemical changes in dairy cattle exposed to chronic, low-level aflatoxins. Although total aflatoxin concentrations in feed (AFLA-T) displayed substantial seasonal variability—from summer lows of 1–7 µg/kg to late-storage peaks exceeding 30 µg/kg in corn, silage, and mixed ration—these fluctuations did not correspond linearly to monthly AFM1 levels in milk. This dissociation is fully consistent with the known nonlinear, saturable, and efficiency-limited nature of the AFB1→AFM1 biotransformation pathway, where hepatic CYP1A2 and CYP3A4 convert only 0.3–6% of ingested AFB1 into AFM1 [47]. The AFM1 concentrations measured here (0.0020–0.0093 μg/kg) are far below the EU maximum limit (0.05 μg/kg), yet they aligned strongly with subtle but structured shifts in biochemical markers, highlighting AFM1 as a sensitive internal biomarker of exposure rather than a mere residue.

The absence of meaningful linear correlations between AFM1 and individual serum parameters (r values −0.15 to +0.26) conforms to the well-established toxicological principle that early aflatoxin hepatotoxicity manifests as diffuse, low-magnitude perturbations rather than dramatic excursions outside reference intervals [42]. Aflatoxins induce mitochondrial dysfunction, oxidative stress, and impairment of hepatocellular protein synthesis long before overt injury becomes detectable [48], which explains the preserved enzymatic values despite subtle shifts across multiple parameters.

The serum profile observed in this work matches classical patterns of compensated hepatic stress. Albumin ranged between 26.7 and 34.9 g/L, and total protein between 68–83 g/L, values consistent with mild suppression of hepatic anabolic function during aflatoxin exposure [43]. ALP showed episodic increases up to 156 U/L toward late winter, reflecting moderate cholestatic or periportal stress. ALT and AST remained within physiological intervals (22–29 U/L and 78–116 U/L), yet displayed seasonal drifts typically associated with oxidative hepatocellular challenge [49]. Triglycerides (2.8–18 mg/dL) and cholesterol (175–216 mg/dL) followed expected metabolic patterns tied to lipid mobilization and hepatic lipid turnover, processes documented in aflatoxin-exposed cattle. Mild increases in creatinine (0.56–0.88 mg/dL) and variable urea values (8–35 mg/dL) are consistent with secondary renal or nitrogen metabolism perturbations described in chronic mycotoxin exposure [50].

Importantly, the multivariate analyses employed here detected coordinated metabolic responses that individual biomarkers alone could not. AFM1 explained over 7% of global biochemical variance in PERMANOVA (*p* = 0.002), showed a strong multivariate effect in MANOVA (Pillai = 0.198, *p* < 6 × 10^−5^), and exhibited a highly significant latent association with biochemical parameters in CCA (*p* < 10^−13^). These patterns reflect predictable biochemical modulation under internal aflatoxin burden and mirror findings from controlled toxicological trials in ruminants and humans [51]. The LDA results further confirmed that animals classified as Borderline—based on small deviations across ALT, AST, GGT, ALP, bilirubin, and total protein—clustered consistently apart from Normal animals, illustrating subtle but coordinated physiological compensation.

Taken together, these findings strengthen the growing consensus that AFM1 is a more accurate biological indicator of aflatoxin exposure than AFLA-T measured in feed. The internal dose, rather than the external contamination level, better predicts metabolic stress, hepatic load, and functional adaptation. This interpretation is fully aligned with contemporary risk assessment frameworks, including recent EFSA consensus documents emphasizing the superiority of internal biomarkers for chronic low-dose exposure scenarios [52] and systematic evaluations of aflatoxin carry-over in dairy cattle [53]. Ultimately, by integrating AFM1 with multivariate biochemical profiling, the present study provides a mechanistically grounded and physiologically sensitive approach for assessing aflatoxin burden, offering practical relevance for dairy herd monitoring under real-world environmental conditions. These findings further support the concept that internal biomarkers such as AFM1 provide a more biologically meaningful measure of aflatoxin burden than external contamination levels measured in feed.

## 4. Conclusions

This integrated ecological, toxicological, and biochemical assessment provides a comprehensive understanding of fungal dynamics, mycotoxin behavior, and AFM1 transfer under real farm conditions in dairy production systems. The results demonstrate that fungal proliferation, secondary metabolite formation, and toxicokinetic outcomes cannot be reliably predicted from fungal biomass or simple linear relationships between feed contamination and milk residues, but rather emerge from complex interactions among moisture availability, substrate characteristics, and environmental instability within feed matrices.

Fungal community structure across feed substrates was shown to be primarily governed by moisture content, acting as the main ecological filter at the genus level. Xerophilic *Aspergillus* dominated dry cereals and premixes, *Fusarium* proliferated in high-moisture substrates such as silage and fibrous feeds, while *Penicillium* occupied intermediate ecological niches. These patterns confirm distinct, substrate-driven ecological strategies with direct implications for mycotoxin risk.

Mycotoxin dynamics displayed a clear dissociation from fungal counts, with OTA and ZEA showing pronounced seasonal variability driven by chemical persistence and microenvironmental stress rather than fungal abundance. AFLA-T followed storage-related dynamics, whereas DON remained comparatively stable. These findings support the interpretation of mycotoxins as stress-induced, substrate-mediated metabolites rather than direct proxies of fungal biomass.

Despite marked fluctuations in feed contamination, AFM1 concentrations in milk remained consistently below 0.01 μg/kg, indicating limited carry-over and effective toxicokinetic buffering under moderate exposure conditions. Weak associations between feed aflatoxins and AFM1, together with minimal changes in serum biochemical markers, reflect a predominantly subclinical exposure scenario.

A limitation of the present study is that the routine inclusion of a mycotoxin-binding additive likely reduced aflatoxin bioavailability, attenuating AFM1 transfer and masking part of the physiological response to contaminated feed. Consequently, the observed effects should be interpreted as reflecting mitigated exposure conditions rather than unbuffered toxicological impact.

Overall, these findings highlight the multifactorial nature of mycotoxin risk in dairy production and underscore the need for integrated monitoring strategies combining feed analysis, milk residue surveillance, and biochemical profiling. Multivariate approaches proved essential for capturing subtle physiological responses and offer a robust framework for managing chronic low-dose mycotoxin exposure under real-world farm conditions.

## 5. Materials and Methods

### 5.1. Study Design and Experimental Setting

The study was conducted throughout 2023 in a commercial dairy farm located in the eastern Moldavian Plateau (Romania), an area with temperate-continental climatic influence. A fixed group of 15 clinically healthy primiparous Romanian Black Spotted cows (n = 15), homogeneous in age (24–27 months), body weight (~700 kg), and milk yield (~24 kg/day), was monitored monthly.

Sampling was fully synchronized: on each sampling day, feed ingredients, TMR, raw milk, and blood were collected from the same 15 cows within a controlled time window. This ensured complete traceability across the feed–animal–milk continuum and allowed accurate monthly pairing between AFLA-T in feed, AFM1 in milk, and biochemical profiles in serum.

### 5.2. Diet Formulation and Nutritional Coverage

Nutritional requirements were established according to INRA standards [54], while the detailed composition of the feed ration is presented in Table 5. The diet incorporated corn, wheat, soybean meal, rapeseed meal, feed premix, salt, yeast culture, calcium supplement, hydrogenated fat, and brewers’ grains, forming a nutritionally balanced formulation designed for high-producing dairy cows.

Based on the ingested dry matter (IDM), the approximate nutrient composition per kilogram included 80 g crude ash, 920 g organic matter, 180 g crude protein, 50 g ether extract, 170 g crude fiber, 220 g acid detergent fiber (ADF), 340 g neutral detergent fiber (NDF), and 330 g non-fibrous carbohydrates (NFC).

Throughout the study, cows received the multi-component mycotoxin-deactivating additive Mycofix^®^ (BIOMIN GmbH, Getzersdorf, Austria) at 10–50 g/animal/day, corresponding to an average inclusion level of approximately 1–2 g/kg feed ration on a dry-matter basis.

Throughout the study, the TMR was supplemented with a commercial mycotoxin-binding additive (Mycofix^®^; BIOMIN/DSM, Austria) at the manufacturer-recommended dose (10–50 g/cow/day), corresponding to ~1–2 g/kg TMR dry matter. The additive does not interfere with ELISA-based AFM1 quantification but may influence gastrointestinal bioavailability of aflatoxins.

### 5.3. Sampling Strategy and Traceability

Feed ingredients (corn, wheat, soybean meal, rapeseed meal, premix, corn silage, alfalfa hay) and TMR were sampled monthly following Regulation (EC) No 152/2009 [55]. For each matrix, ten composite samples were obtained from ≥5 incremental subsamples collected along the active silage face or feed batch.

Milk samples were collected at the morning milking, and blood samples were collected 4–6 h after feeding by coccygeal venipuncture. All samples were coded by animal and by feed batch, enabling direct monthly matching between exposure indicators (AFLA-T, OTA, ZEA, DON), milk AFM1, and serum biomarkers.

### 5.4. Moisture, Fungal Load, and Mycotoxin Analyses

Moisture content was determined gravimetrically using the ISO 6496:1999/SR ISO 6496:2001 protocol [56]. Approximately 2–3 g of homogenized sample were dried to constant mass at 105 °C for 4–5 h in a thermostatic oven (POL-EKO SLW 115 TOP+, POL-EKO sp.k., Wodzislaw Slaski, Poland). Weights were recorded using an analytical balance Radwag XA 82/220.3Y (Radwag, Radom, Poland; reproducibility ±0.1 mg). Quality assurance included duplicate analyses with acceptance criteria of ≤0.5% difference between replicates and daily verification of oven temperature using a traceable thermometer.

### 5.5. Fungal Enumeration and Taxonomic Identification

#### 5.5.1. Total Fungal Load (CFU/g)

Fungal counts were assessed using decimal dilutions (10^−1^–10^−6^) prepared in saline supplemented with 0.1% Tween-80. One gram of each feed matrix was suspended in 9 mL diluent and homogenized using a Heidolph Top-Mix Evolution vortex (Heidolph, Schwabach, Germany). Double-layer PDA plates (potato dextrose agar enriched with 100 mg/L chloramphenicol) were inoculated and incubated at 25–27 °C. Colony enumeration was performed on days 3 and 5, following EN ISO 16140-2:2016 guidelines. Results were expressed as CFU/g [57].

#### 5.5.2. Morphological Identification

Fungal identification was performed at the genus level based on morphological characteristics, which does not allow discrimination of individual toxigenic species; species-level resolution would require molecular approaches.

Representative fungal colonies were subcultured on PDA for 5–7 days and examined macroscopically and microscopically. Microscopic evaluation was performed using a Motic DMWB1-223 ASC optical microscope (Motic, Barcelona, Spain), equipped with 10× to 100× objectives. Genus-level classification followed established taxonomic keys (Leslie & Summerell for *Fusarium*; Pitt & Hocking for *Aspergillus* and *Penicillium*) [25,58]. Colonies with uncertain morphology were re-evaluated 24–48 h later, with identification restricted to the genus level to ensure consistency.

### 5.6. Mycotoxin Analysis in Feed

Aflatoxins (AFLA-T: AFB1 + B2 + G1 + G2), ochratoxin A (OTA), zearalenone (ZEA), and deoxynivalenol (DON) were quantified using competitive direct ELISA kits (AgraQuant^®^, Romer Labs, Tulln, Austria). Feed extraction involved mixing 20 g of homogenized sample with 100 mL of 70% methanol (1:5 m/v), vortexing for 3 min, and filtering through Whatman No. 1 paper; DON extraction was performed in aqueous medium with subsequent 1:4 dilution.

Absorbances were measured at 450 nm with 630 nm reference using a Stat Fax^®^ 2100 microplate reader (Awareness Technology, Palm City, FL, USA). The 4-parameter logistic (4-PL) model was used for quantification. Only results with R^2^ ≥ 0.99, duplicate CV ≤ 15%, and acceptable internal controls were validated. Mycotoxin concentrations were reported as ppb (AFLA-T, OTA, ZEA) or mg/kg (DON), and results were interpreted in relation to European regulatory benchmarks (Reg. 574/2011; Rec. 2016/1319) [25,59].

### 5.7. Determination of AFM1 in Milk

AFM1 was quantified using the AgraQuant^®^ Aflatoxin M1 High Sensitivity kit (5–100 ng/kg) produced by RomerLabs, Tulln, Austria). Milk samples were centrifuged at 3000 rpm for 10 min, and 100 µL lactoserum was analyzed. Absorbance was recorded using an MR-96A ELISA reader (Mindray, Shenzhen, China). The 4-PL calibration curve was applied, and results were expressed as ng/kg and converted to ppb. Compliance assessment was based on the EU maximum permissible level of 50 ng/kg (Reg. 915/2023) [60].

### 5.8. Serum Biochemistry

Blood was collected monthly from the coccygeal vein using 16 G × 1½″ sterile needles and 9 mL clot-activator tubes (MasterLab^®^, Beijing, China). Samples were centrifuged at 3000 rpm for 5 min using a Hettich Rotofix 32A centrifuge (HettichLab, Tuttlingen, Germany). Serum biochemical parameters—including ALT, AST, GGT, ALP, total protein, albumin, bilirubin, cholesterol, triglycerides, urea, creatinine, and calcium—were measured using a BioSystems BA200 automated analyzer (BioSystems S.A., Barcelona, Spain). Analytical reliability was ensured through daily automatic calibration and internal quality controls according to ISO 15189:2022 [61].

### 5.9. Statistical Analysis

All raw data were initially compiled and validated in Microsoft Excel prior to statistical processing. Subsequent analyses were performed in RStudio (R version 4.4.1; R Foundation for Statistical Computing, Vienna, Austria) and SPSS Statistics v.21.0 (IBM Corp., Armonk, NY, USA). Additional graphical analyses were produced in GraphPad Prism v.10.0 (GraphPad Software, San Diego, CA, USA).

Prior to inferential testing, all variables were screened for outliers, missing values, and adherence to distributional assumptions. Normality was assessed using the Shapiro–Wilk test in combination with visual inspection of Q–Q plots and histograms.

Univariate monthly comparisons of mycotoxins, fungal load, AFM1, and biochemical parameters were performed using one-way ANOVA for normally distributed variables, followed by LSD post hoc testing. Variables deviating from normality were analyzed using the Kruskal–Wallis test with Dunn–Bonferroni correction. Data are expressed as mean ± SD, with statistical significance defined at *p* < 0.05 (*) and *p* < 0.01 (**).

Correlation analyses were conducted based on distributional properties: Pearson’s correlation coefficient (r) was applied to variables showing approximate normality after screening and normalization, while Spearman’s rank correlation (ρ) was used for non-normally distributed data. Associations were evaluated between: (i) moisture content and fungal load (CFU/g); (ii) fungal load and mycotoxin concentrations in feed; (iii) feed mycotoxins and AFM1 in milk; and (iv) AFM1 and serum biochemical markers.

Multivariate data structure was explored using several complementary approaches implemented in R. Principal Component Analysis (PCA) was conducted on standardized biochemical variables to extract PC1–PC3 for subsequent modeling. Multivariate Analysis of Variance (MANOVA) was used to evaluate the contribution of AFM1 or total aflatoxins (AFLA-T) to variance within the biochemical profile, using Pillai’s Trace and Wilks’ Lambda. PERMANOVA (adonis2, vegan package; 999 permutations; Euclidean distance) was applied as a distribution-free multivariate test to quantify the proportion of biochemical variance explained by exposure classes. Canonical Correlation Analysis (CCA) was employed to explore latent relationships between the aflatoxin block (AFLA-T, AFM1) and the biochemical block (ALT, AST, ALP, GGT, PROT-T, ALB, CREA, URE, COL, TRIG, Ca).

Linear Discriminant Analysis (LDA) was performed using the MASS package to classify animals into Normal versus Borderline hepatic status, defined according to the number of biochemical parameters outside physiological ranges. LDA projections onto LD1 were visualized using 95% confidence ellipses and density overlays (ggplot2, ggforce).

Graphical outputs—including heatmaps, correlation matrices, PCA biplots, LDA scatterplots, and LD1 density distributions—were generated in R using ggplot2, pheatmap, corrplot, and base packages. All analyses followed standardized and reproducible workflows implemented in RStudio (R v4.4.1).

## Figures and Tables

**Table 1:** Mycological analysis of feedstuffs used in dairy cattle nutrition (×10^3^ CFU/g). Values are expressed as mean ± SD. Number of samples indicates the number of samples per feed type (N = 12); each sample was analyzed in triplicate (n = 3).

Sample	N	*Aspergillus* spp.	*Penicillium* spp.	*Fusarium* spp.	Other Fungi	Total Fungi	Moisture (%)
Corn	12	3.03 ± 1.63	1.56 ± 0.91	1.76 ± 1.12	0.48 ± 0.54	6.82 ± 2.18	13.36 ± 0.69
Wheat	12	2.03 ± 1.73	1.72 ± 1.22	1.73 ± 1.42	0.83 ± 0.83	6.31 ± 2.70	13.16 ± 0.85
Feed premix	12	3.32 ± 1.93	1.06 ± 1.21	2.15 ± 1.67	0.50 ± 0.78	7.04 ± 2.52	11.27 ± 0.99
Soybean meal	12	2.46 ± 1.59	1.11 ± 0.85	2.55 ± 2.09	0.32 ± 0.44	6.43 ± 2.99	11.43 ± 0.64
Rapeseed meal	12	1.96 ± 1.36	1.19 ± 0.95	2.06 ± 1.49	0.17 ± 0.34	5.38 ± 1.99	9.97 ± 0.85
Feed ration	12	2.84 ± 1.55	1.37 ± 0.93	2.44 ± 1.50	0.52 ± 0.39	7.17 ± 2.33	54.28 ± 2.56
Corn silage	12	2.92 ± 1.66	1.72 ± 1.22	2.22 ± 1.50	0.64 ± 0.45	7.51 ± 2.47	61.40 ± 4.05
Alfalfa hay	12	1.92 ± 1.19	1.33 ± 0.89	2.18 ± 1.32	0.38 ± 0.35	5.81 ± 2.18	11.89 ± 1.21

**Table 2:** Significant correlations (*p* < 0.05) between moisture content (%) and fungal load (CFU/g) of major fungal genera and total fungi in feed matrices.

Feed Matrix	*Aspergillus*	*Penicillium*	*Fusarium*	Other spp.	Total Fungi
Corn	–	–	0.157 *	0.172 **	–
Wheat	−0.221 **	−0.146 *	0.558 **	0.387 **	0.241 **
Feed premix	−0.191 **	−0.416 **	0.473 **	0.508 **	0.159 *
Soybean meal	–	–	−0.525 **	−0.134 *	−0.464 **
Rapeseed meal	0.293 **	–	−0.362 **	−0.173 **	–
Feed ration	0.173 **	–	0.162 *	–	0.254 **
Corn silage	–	–	0.190 **	–	–
Alfalfa hay	0.260 **	0.145 *	0.277 **	0.173 **	0.398 **

* Moderate positive or negative correlation; ** Strong positive or negative correlation.

**Table 3:** Significant inter-genera correlations across feed types (*p* < 0.05) (Only statistically significant correlations shown).

Fungal Pair	Corn	Wheat	Feed Premix	Soybean Meal	Rapeseed Meal	Feed Ration	Corn Silage	Alfalfa Hay
*Aspergillus*—Total fungi	0.660 **	0.466 **	0.630 **	0.623 **	0.402 **	0.688 **	0.587 **	0.526 **
*Fusarium*—Total fungi	0.587 **	0.494 **	0.543 **	0.760 **	0.640 **	0.628 **	0.566 **	0.754 **
*Penicillium*—Total fungi	—	0.570 **	—	0.337 **	0.312 **	0.267 **	0.463 **	0.429 **
*Fusarium*—Other spp.	0.228 **	0.249 **	0.362 **	0.161 *	0.253 **	—	—	0.199 **
*Aspergillus*—*Fusarium*	—	−0.228 **	—	0.131 *	−0.210 **	0.212 **	—	0.233 **
*Penicillium*—*Fusarium*	—	—	−0.382 **	—	−0.169 **	−0.168 **	—	—
*Aspergillus*—Other spp.	—	−0.201 **	−0.293 **	−0.309 **	−0.397 **	−0.198 **	—	—

* Moderate positive or negative correlation; ** Strong positive or negative correlation.

**Table 4:** Summary of multivariate statistics used to evaluate the association between aflatoxin exposure (AFLA-T, AFLA-M1) and biochemical response.

Analysis	Metric	Result
Pearson/Spearman correlations	r range	−0.15 to +0.26
PERMANOVA (AFLA-T categories)	F = 1.73, R^2^ = 0.033, *p* = 0.115	Not significant
PERMANOVA (AFLA-M1 categories)	F = 8.01, R^2^ = 0.073, *p* = 0.002	Significant
CCA—Canonical correlation 1	*p* < 1 × 10^−13^	Highly significant
MANOVA—PC1–PC3 vs. AFLA-M1	Pillai = 0.198, F = 8.24, *p* = 5.9 × 10^−5^	Significant
MANOVA—PC1–PC3 vs. AFLA-T	Pillai = 0.113, *p* = 0.068	Marginal
LDA Variant C (Hepatic Status)	Dominant LD1 loadings = PC3 (−0.57), PC1 (−0.49)	Biochemistry-driven separation
LDA Classification Balance	Normal = 92–93%; Borderline = 7–8%	Strong class imbalance
LD1 distribution (Figure 7B)	Normal → LD1 > 0; Borderline → LD1 < 0	Partial overlap

**Table 5:** Daily nutritional requirements and TMR coverage for lactating dairy cows (700 kg BW, 24 kg milk/day, 4% fat).

Parameter	Requirement	TMR Provided
DMI (kg/day)	19.5	20.0
Milk Feed Units (MLD_U)	17.1	16.7
Net Energy for Milk (NEM_U)	16.6	17.6
PDI-N (g/day)	1645	1720
PDI-E (g/day)	1645	1660
Calcium (g/day)	136	135
Phosphorus (g/day)	76	77

Abbreviations: TMR, total mixed ration; DMI, dry matter intake; MLD_U, milk feed units; NEM_U, net energy for milk; PDI-N, protein digestible in the intestine limited by nitrogen supply; PDI-E, protein digestible in the intestine limited by energy supply; BW, body weight.

## Data Availability

The datasets presented in this article are not readily available because the data are part of an ongoing study related to a PhD thesis. Requests to access the datasets should be directed to Ioana Poroșnicu (ioana.porosnicu@yahoo.com).

## Abbreviations

The following abbreviations are used in this manuscript:

AFLA-T	Total Aflatoxins
OTA	Ochratoxin A
AFM1	Aflatoxin M1
ZEA	Zearalenone
DON	Deoxynivalenol
PDA	Potato Dextrose Agar
